# Exploring the mechanism by which aqueous *Gynura divaricata* inhibits diabetic foot based on network pharmacology, molecular docking and experimental verification

**DOI:** 10.1186/s10020-023-00605-w

**Published:** 2023-01-20

**Authors:** Yu Sun, Cailiang Gao, Huiting Liu, Xue Liu, Tun Yue

**Affiliations:** Chongqing University Three Gorges Hospital, No. 165 Xincheng Road, Wanzhou District, 404000 Chongqing, People’s Republic of China

**Keywords:** *Gynura divaricata*, Network pharmacology, Diabetic foot, Molecular docking

## Abstract

**Background:**

To predict and validate the potential mechanism by which *Gynura divaricata* (GD) functions in the treatment of diabetic foot (DF).

**Methods:**

The main chemical constituents of GD were identified by reviewing the literature, the traditional Chinese medicine database platform (TCMIP) and the BATMAN-TCM platform. DF disease targets were identified with the GeneCards database, and the compound-target network was constructed by using the intersection of drugs and disease. The STRING platform was used to construct the protein–protein interaction (PPI) network, and Cytoscape 3.7.2 software was used to visualize the results. Moreover, the Metascape database was used for Gene Ontology (GO) enrichment analyses and Kyoto Encyclopedia of Genes and Genomes (KEGG) enrichment analyses. Molecular docking of the active ingredients of GD and core protein targets of DF was performed using AutoDock software. Finally, the predicted results were preliminarily verified with experiments.

**Results:**

A total of 140 potential targets of GD were identified and associated with DF. According to the PPI network analysis, GD accelerated DF wound healing, and the mechanism may be related to proteins such as AKT1, TP53, IL6, CASP3, TNF, and VEGFA. GO and KEGG enrichment analyses indicated that GD may play a role in the treatment of diabetic foot by affecting various signaling pathways. Molecular docking results showed that the proteins AKT1, TP53, IL6, CASP3, TNF, and VEGFA were closely associated with the components of GD. The animal experiments showed that GD reduced the levels of IL-6 and TNF-α and increased the mRNA and protein expression of VEGFA in rats with DF.

**Conclusions:**

GD regulates multiple targets and multiple pathways to promote wound healing in DF.

**Supplementary Information:**

The online version contains supplementary material available at 10.1186/s10020-023-00605-w.

## Introduction

Diabetic foot (DF) is a common chronic complication of diabetes (Pop-Busui et al. 2021); DF causes substantial physical and psychological pain for patients, places a great economic burden on patients, and seriously threatens people's health. Ischemia is a substantial aspect of the pathology of diabetic foot that leads to the characteristic feature of foot ulceration. The cellular and molecular mechanisms underlying the pathogenesis and prevention of DF are insufficiently understood. DF can be complicated by infection and may eventually result in amputation (Edmonds et al. 2021). These features contribute to the considerable clinical and economic burdens associated with DF. Therefore, the clinical treatment of DF is still an urgent problem that needs to be solved.

Traditional Chinese medicine has a long history of using traditional herbs to treat diabetes. *Gynura divaricata* (GD) is a perennial herb that is native to Singapore and is now abundant in the southwestern and southeastern coastal areas of China (Xu and Zhang 2017). GD is a natural plant that can be used for both medicine and food and has broad application prospects. Studies have shown that it has various pharmacological effects, including hypoglycemic (Li et al.^2009^; Wu et al. 2011; Xu et al.^2015^), antitumor (Ashraf et al.^2020^; Yi et al. 2016), blood pressure-lowering (Hong et al. 2020) and antioxidative stress effects (Wan et al.^2011^; Dong et al.^2019^). Xu et al. (2015) indicated that the hypoglycemic activities of GD improved antioxidant capacity and insulin signaling in mice with type 2 diabetic. Investigations have also shown that GD polysaccharides and extracts have antihyperglycemic effects on rats with type 2 diabetes. These studies successfully showed that GD is a potentially effective drug for the treatment of diabetic foot. However, the detailed mechanisms remain unclear. Based on the good efficacy of GD in the treatment of diabetes, we designed a study to explore whether GD can ameliorate the symptoms of DF and to elucidate the specific mechanism.

Network pharmacology studies the mechanisms of drug action at the overall level, and it can provide a reliable theoretical basis for understanding the complex mechanism by which traditional Chinese medicine regulates multiple targets and multiple pathways (Zhou et al.^2020^). Traditionally, Chinese medicine (TCM) has been used to treat diabetes mellitus in China for more than 2000 years (Ji et al. 2013). However, an understanding of the main pharmacological activities of herbal medicines from a systems pharmacology perspective remains elusive, which hinders the development of modern herbal medicines. Network pharmacology provides a novel method for analyzing traditional Chinese medicine by accurately identifying the targets of multiple components. The increasing understanding of pharmacology has led to wide acceptance of the “multitarget, multidrug” model instead of the “one target, one drug” model.

In this study, the network pharmacology method was used to construct a protein–protein interaction network of the shared targets of GD and DF. Then, screening was performed to identify the hub genes of the protein–protein interaction (PPI) network. In addition, the core targets were used to study the target and target pathway of the active ingredients of GD by using network pharmacology, gene ontology (GO), and biological pathway (KEGG) functional enrichment analyses. Finally, we performed a molecular docking analysis and some experiments to verify our predictions.

## Materials and methods

### Prediction and identification of components and targets of GD

**Fig. 1:**
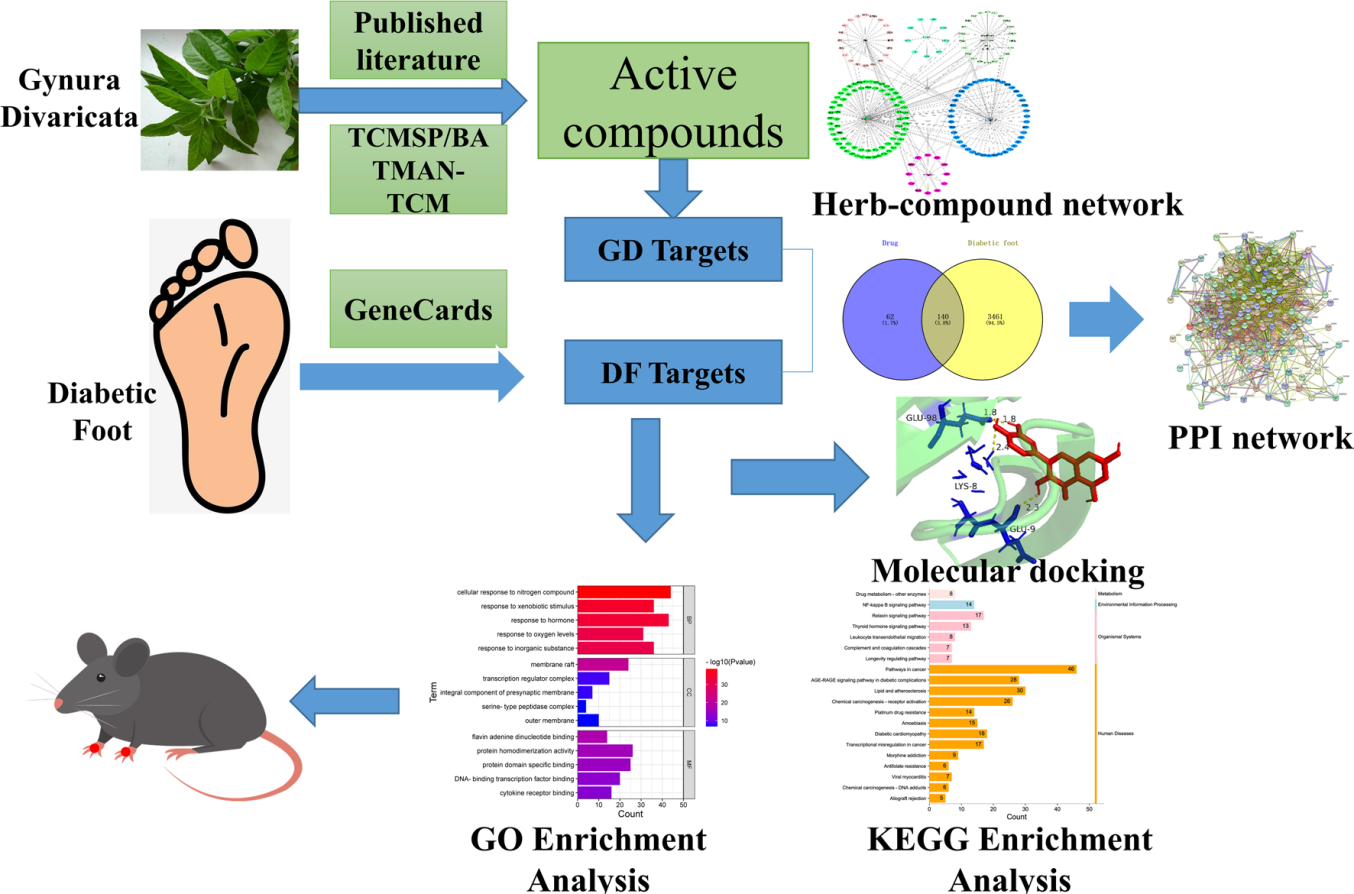
Workflow of the systematic strategies based on network pharmacology

Figure 1 provides a graphical summary of the experimental workflow used in this study. The components of each herb in GD were obtained from TCMSP (http://tcmspw.com/), BATMAN-TCM (http://bionet.ncpsb.org.cn/batman-tcm/), and published literature (Xu and Zhang 2017; Chou et al. 2012; Chen et al. 2010). Targets were identified in the TCMSP database and BATMAN-TCM database according to the criteria of OB ≥ 30, DL ≥ 0.18 and Score cutoff > 20, *P* < 0.05, respectively. The protein name was converted into Gene Name through the UniProt platform (https://www.uniprot.org/).

### DF target screening and identification

The Genecards database (https://www.genecards.org/) was searched to identify potential disease-related targets by using “Diabetic Foot” as a keyword. According to experience, target points with a Z-Socre > 1.0 were selected as potential targets in DF.

### Construction of the intersection network and PPI network

A Venn diagram was used to identify the screened targets that were shared by GD and DF. The common targets were considered the therapeutic targets of GD in the treatment of DF, as described previously (Liang et al. 2021). The STRING database (https://cn.string-db.org/) was used to construct a target protein–protein interaction (PPI) network (Szklarczyk et al. 2019). By setting *P* > 0.4, a network of protein interactions was generated. The PPI network was visually analyzed by Cytoscape 3.7.2 software (Shannon et al. 2003).

### Biology functional analysis

Gene Ontology (GO) and Kyoto Encyclopedia of Genes and Genomes (KEGG) describe and annotate screened genes and contribute to the interpretation of system-level data (Zhou et al. 2019). Metascape (https://metascape.org/) was used to further explore the complex mechanism by which GD functions in the treatment of DF. Enrichment results were considered significant at *P* < 0.01, and the analysis results were visualized.

### Verification by molecular docking

The core protein structure of the PPI network was downloaded from the PDB database (http://www.rcsb.org/), and PyMOL software was used to remove ligands and water molecules. The.sdf file of the active ingredients of GD was downloaded from the PubChem database (https://pubchem.ncbi.nlm.nih.gov/). Autodock Tools 1.5.6 software was used to add atomic charges and assign atomic types. All the flexible bonds were set as rotatable Running AutoDock Vina for molecular docking. The lower the energy is when the ligand–receptor interaction conformation is stable, the more likely the effect is to occur.

### Animal experiment and treatments

Eighty SPF grade healthy male SD rats were purchased from the Animal Experimental Center of Chongqing University Three Gorges Hospital. The animal experimental protocol was performed under an animal license issued by the Health Department of the Government of China and was approved by the Experimental Animal Administrative Committee of Chongqing Three Gorges Central Hospital.

Eight rats were randomly assigned to the control group, and the rest were used to establish the model. The model group was fed a high-fat diet for 4 weeks and then intraperitoneally injected with 1% STZ (S0130; Sigma Merck) solution (40 mg·kg-1) one time. Intravenous blood was collected to monitor fasting blood glucose levels ≥ 16.7 mmol/L, and polydipsia, polyphagia and polyuria were considered indications that the diabetes model had been successfully established (Srinivasan et al. 2005). Then, a stamp was used to make a rectangular mark (3 mm × 7 mm) on the corresponding dorsum of the foot, and the full-thickness skin marked by the rectangle was removed to establish a model of diabetic foot with lower extremity ischemia. Forty rats in which the model was successfully established were randomly selected and divided into a DF model group and a GD group. The GD group was administered 16 g/kg/d, 8 g/kg/d and 4 g/kg/d GD by gavage, and the DF group and the previous control group were given the same volume of normal saline gavage. On the 1st, 4th, 7th, 14th, and 21st days, the wounds on the feet of the rats were photographed, and the wound area was calculated by the following formula: healing rate = (healing area/original wound area) × 100%. After 4 weeks of treatment, blood was collected from the common carotid artery after pentobarbital anesthesia.

### Measurement of inflammatory factors

The levels of the inflammatory factors IL-6 and TNF-α in the serum were determined by ELISA (Neobioscience, China), and the specific steps were carried out according to the instructions of the kit.

### Western blotting

Total protein was extracted and collected from ischemic foot tissue, and the concentration of the total protein was measured. The proteins were separated by 8% SDS–polyacrylamide gel electrophoresis and transferred to PVDF membranes. After blocking in 5% skim milk powder for 2 h at room temperature, the membranes were incubated with primary antibodies overnight at 4 °C and a secondary antibody for 1 h the next day. After washing, the proteins were detected using the ECL Plus chemiluminescence system. The blots were analyzed using ImageJ, and protein expression levels were normalized to the GAPDH expression levels.

### Quantitative real-time PCR

Briefly, total RNA was extracted using TRIzol reagent (TaKaRa, Japan), and reverse transcription was conducted using RevertAid™. Messenger RNA expression levels were determined by RT–PCR using the SYBR GreenPremix Ex TaqTM kit (ToYoBo, Japan) following the manufacturer's instructions. GAPDH was used as an internal control. The 2^−ΔΔCt^ method was used to evaluate fold changes in expression relative to the control. The primers specific for the target genes are listed in Additional file 1: Table S1.

### Statistical analysis

SPSS 17.0 statistical software was used for statistical analysis. The experimental data are expressed as the mean ± SD. One-way ANOVA was used for comparisons between groups. The LSD method was used for comparisons between groups. For data with a non-normal distribution or uneven variance, a nonparametric test of multiple independent samples was used. *P* < 0.05 indicates that the difference is statistically significant.

## Results

### Screening of GD targets and DF disease targets

**Fig. 2:**
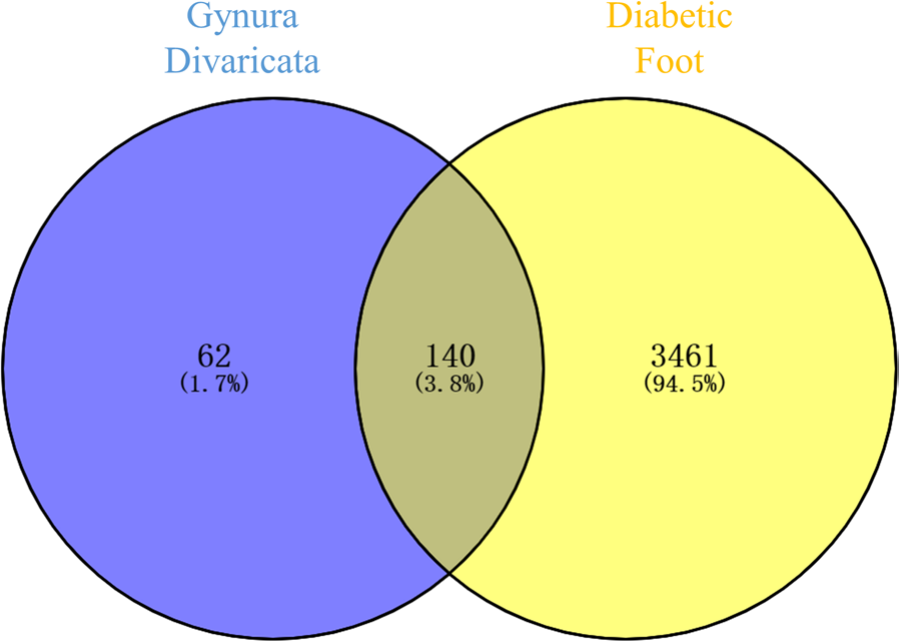
Target Venn diagram of GD and DF

Searches of the TCMSP and BATMAN-TCM databases and published literature revealed that GD contains 25 traditional Chinese medicine active ingredients. Among these active ingredients, 17 compounds had no known potential targets, while 8 compounds had many targets. Some molecules are targets of many kinds of compounds (Additional file 1: Table S2), and 202 targets were identified after removing duplicates. DF disease targets were identified by searching the GeneCards databases. After merging and deleting duplicate targets, a total of 3601 disease targets remained. By overlapping the predicted targets of GD and DF with a Venn diagram, we ultimately identified 140 potential targets of GD in the treatment of DF (Fig. 2).

### Construction of a drug-active ingredient-target network of GD in the treatment of diabetic foot

**Fig. 3:**
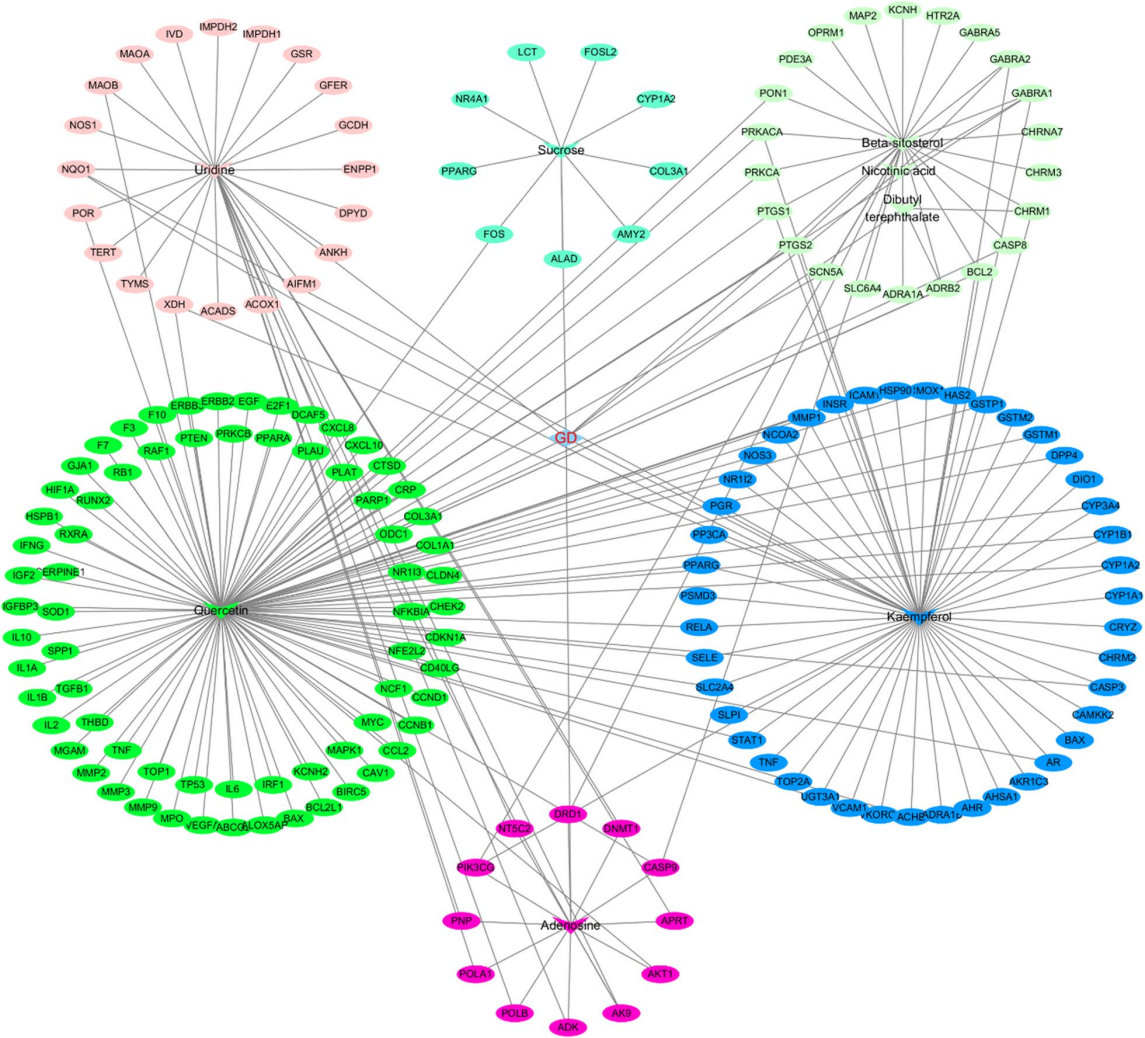
Herb-Compound-Targets network. The inverted V-shaped pattern is the drug molecule, and the ellipse-shaped is the drug targets, and its color is the same as the color of the component it belongs to

To understand the multicomponent pharmacological mechanisms underlying the effects of GD, we constructed an herb-compound-target (H-C-T) network. Information about drug components and effective targets as well as other information about GD was imported into Cytoscape 3.7.2 software to construct a drug-active ingredient-effective target network diagram (Fig. 3). This network consisted of 153 nodes and 180 edges. The compounds with the highest degree values were quercetin, uridine and beta-sitosterol. These compounds may be the key compounds for the treatment of DF.

### Construction of the PPI network

**Fig. 4:**
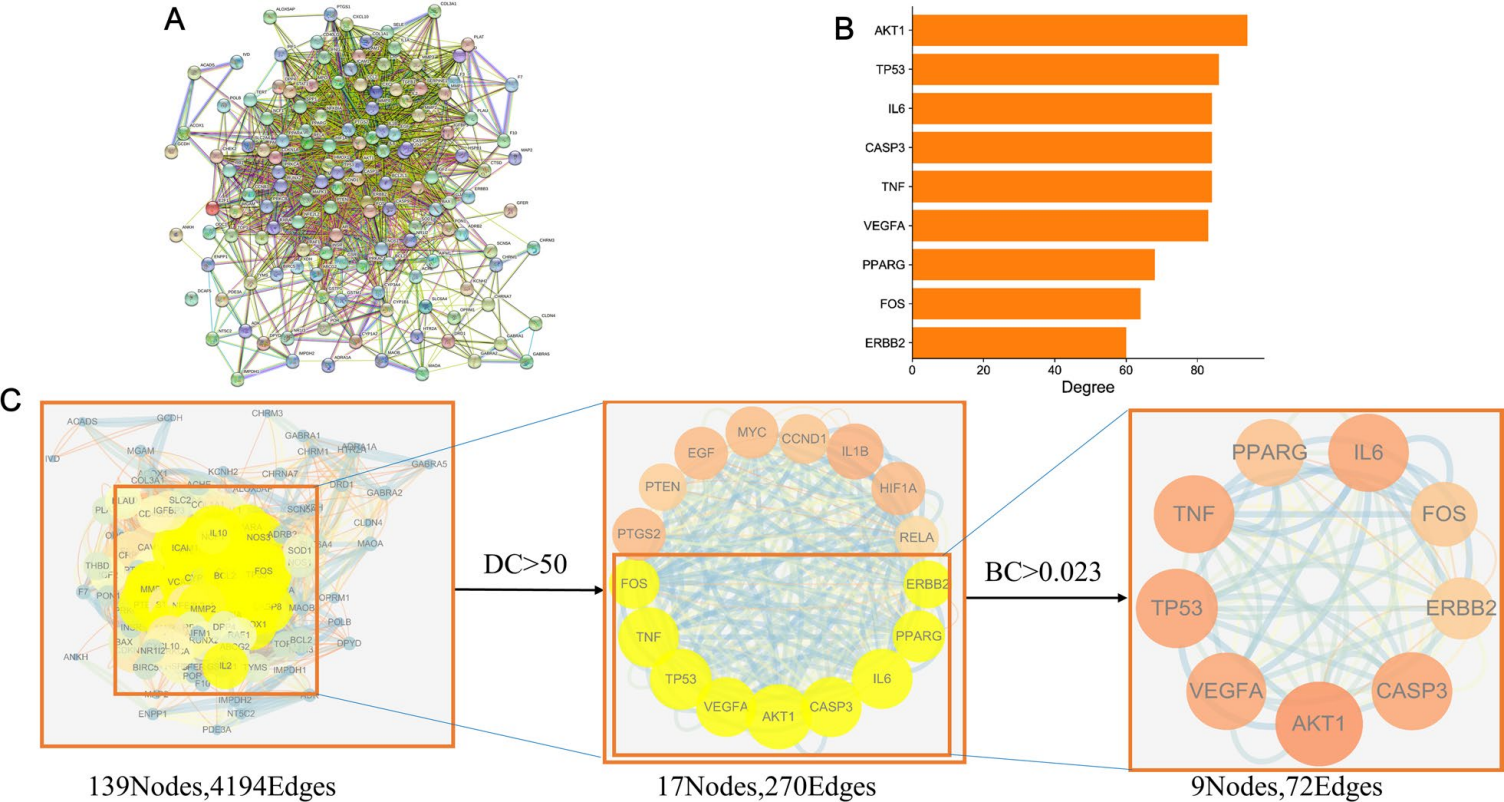
Identification of core targets for GD against DF. **A** Target protein interaction network (PPI). **B** The top 9 hub genes histograms. **C** The process of topological screening for the PPI network. The yellow nodes represent the core targets, and the other nodes represent the noncore targets

In further analysis, the 140 targets identified by the intersection described above were uploaded to the String database (Additional file 1: Table S3), and the species "*Homo sapiens*" was selected to construct a PPI network (Fig. 4A). The downloaded file was imported into Cytoscape to calculate the degree value and reconstruct the PPI network. The network included 139 key nodes and 4196 edges, and the highest node value was 94. Degree value and betweenness centrality (BC) were used to perform the screening. The top core genes were used to generate as a histogram (Fig. 4B). The screening process is shown in Fig. 4C. The top 6 core network proteins that were identified included AKT1, TP53, IL6, CASP, TNF and VEGFA.

### GO and KEGG pathway enrichment analysis

**Fig. 5:**
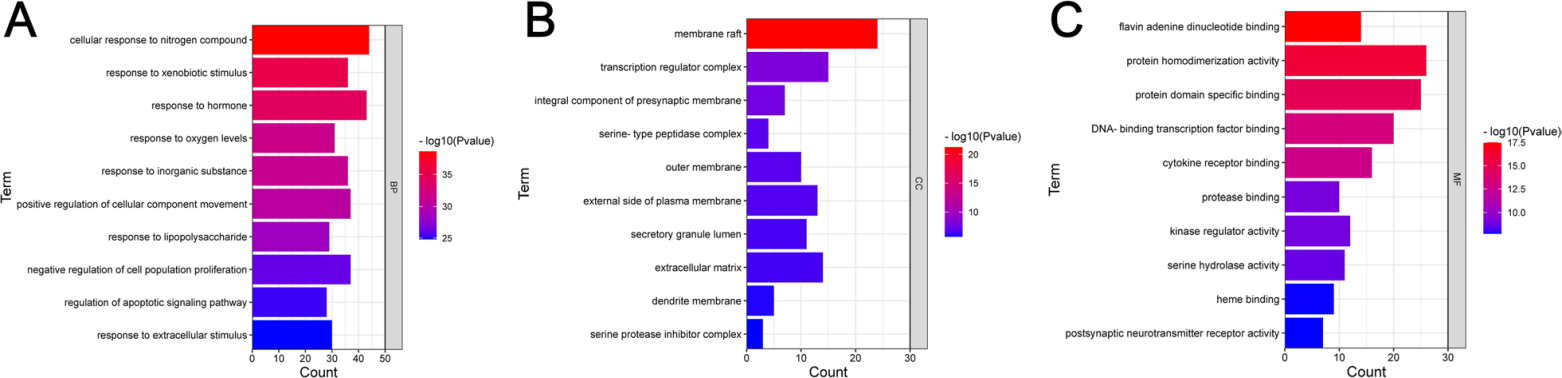
GO enrichment analysis. **A** Biological Process. **B** Cell Component. **C** Molecular Function

**Fig. 6:**
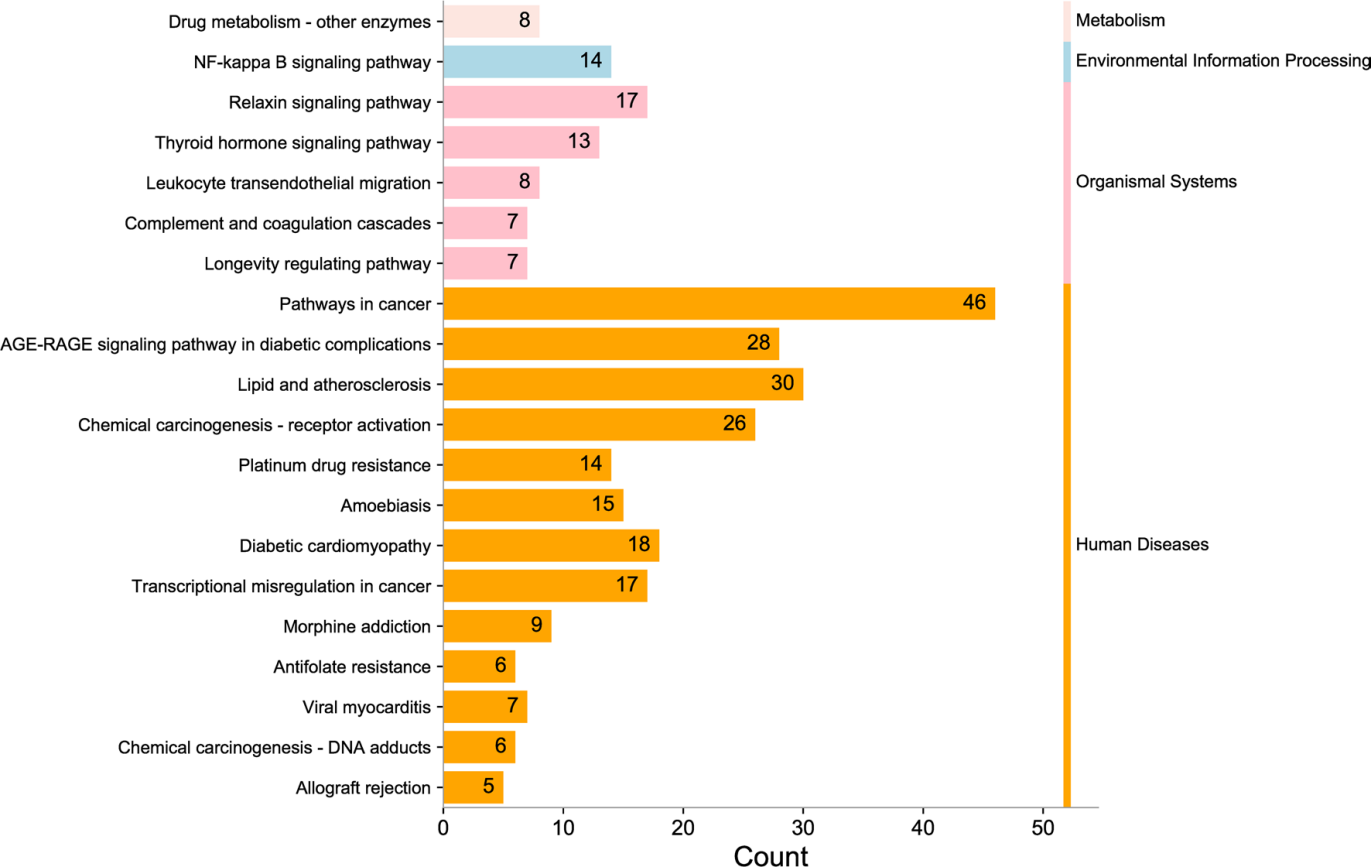
KEGG pathway enrichment analysis

GO and KEGG enrichment analyses of the 140 common targets were performed using Metascape. A total of 516 significantly related GO enrichments were screened, including 272 biological processes (BP), 106 cellular components (CC), and 138 molecular functions (MF). The results were ranked according to *P* < 0.01, count > 3 and Rich factor > 1.5, and 20 significantly enriched BP, CC and MF were identified. The top 10 enriched items are shown in Fig. 5A–C. According to the aforementioned method, the top 20 pathway enrichment results were determined. Subsequently, these pathways were classified and summarized according to KEGG pathway analysis, and the results are shown in Fig. 6. These pathways mainly include pathways in cancer, the AGE-RAGE signaling pathway in diabetic complications, lipids and atherosclerosis, and the most relevant pathway in DF disease is the AGE-RAGE signaling pathway in diabetic complications.

### Molecular docking

**Fig. 7:**
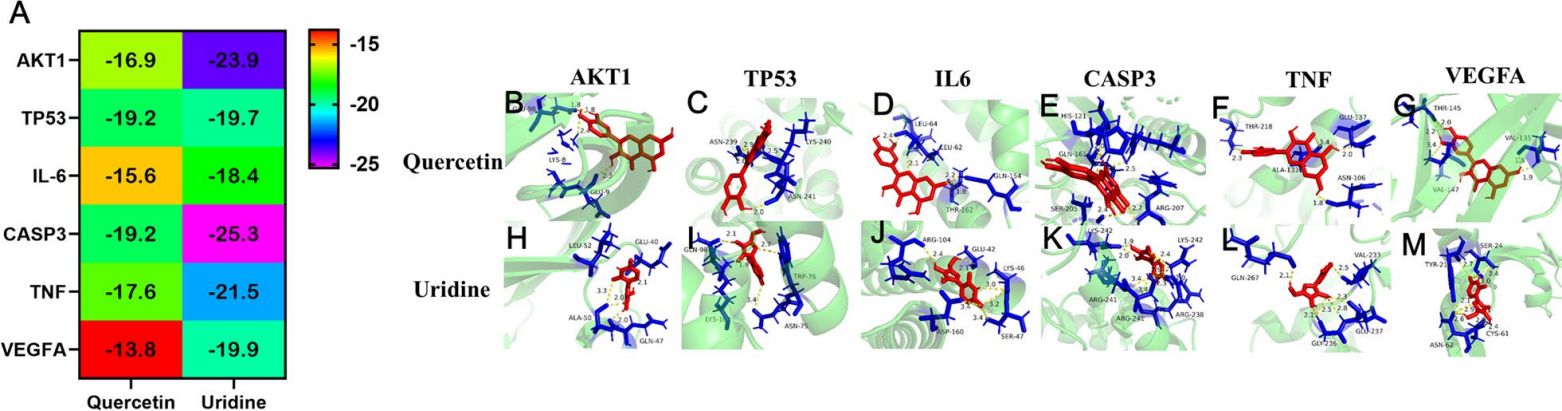
Molecular binding mode diagram. **A** The results of binding energy. **B**–**G** Molecular docking results of Quercetin and core protein. **H**–**M** Molecular docking results of Uridine and core protein

We selected the key genes AKT1, TP53, IL-6, CASP3, TNF-α and VEGEA that were identified in the previous steps and carried out molecular docking with uridine and quercetin, the main pharmaceutical components in GD. The docking energy results are shown in Fig. 7A. The results suggested good docking activity (binding energy less than 1.2 − kcal/mol). All the components mainly interacted with the corresponding targets through hydrogen bonds. Quercetin interacted with GLU-9, LYS-8 and GLU-98 of AKT1 (Fig. 7B). Quercetin interacted with ASN-239, ASN241 and LYS-240 of TP53 (Fig. 7C). Quercetin interacted with LEU-64, LEU-62, THR-162 and GLN-154 of IL-6 (Fig. 7D). Quercetin interacted with HIS-121, GLN-161, SER-205 and ARG-207 of CASP3 (Fig. 7E). Quercetin interacted with THR-218, GLU-137, ALA-133 and ASN-106 of TNF-α (Fig. 7F). Quercetin interacted with THR-145, VAL-135 and VAL-147 of VEGFA (Fig. 7G). Uridine interacted with LEU-52, GLU-40, GLN-47 and ALA-50 of AKT1 (Fig. 7H). Uridine interacted with GLN-98, TRP-76, LYS-168 and ASN-75 of TP53 (Fig. 7I). Uridine interacted with ARG-104, GLU-42, LYS-46, ASP-160 and SER-47 of IL-6 (Fig. 7J). Uridine interacted with LYS-242, ARG-241 and ARG-238 of CASP3 (Fig. 7K). Uridine interacted with GLN-267, VAL-233, GLY-26 and GLU-237 of TNF-α (Fig. 7L). Uridine interacted with TYR-21, SER-24, ASN-62 and CYS-61 of VEGFA (Fig. 7M). The abovementioned ligands could be well embedded in the active pockets of the receptor target proteins.

### GD extract accelerates wound healing in rats with DF at different time points

**Fig. 8:**
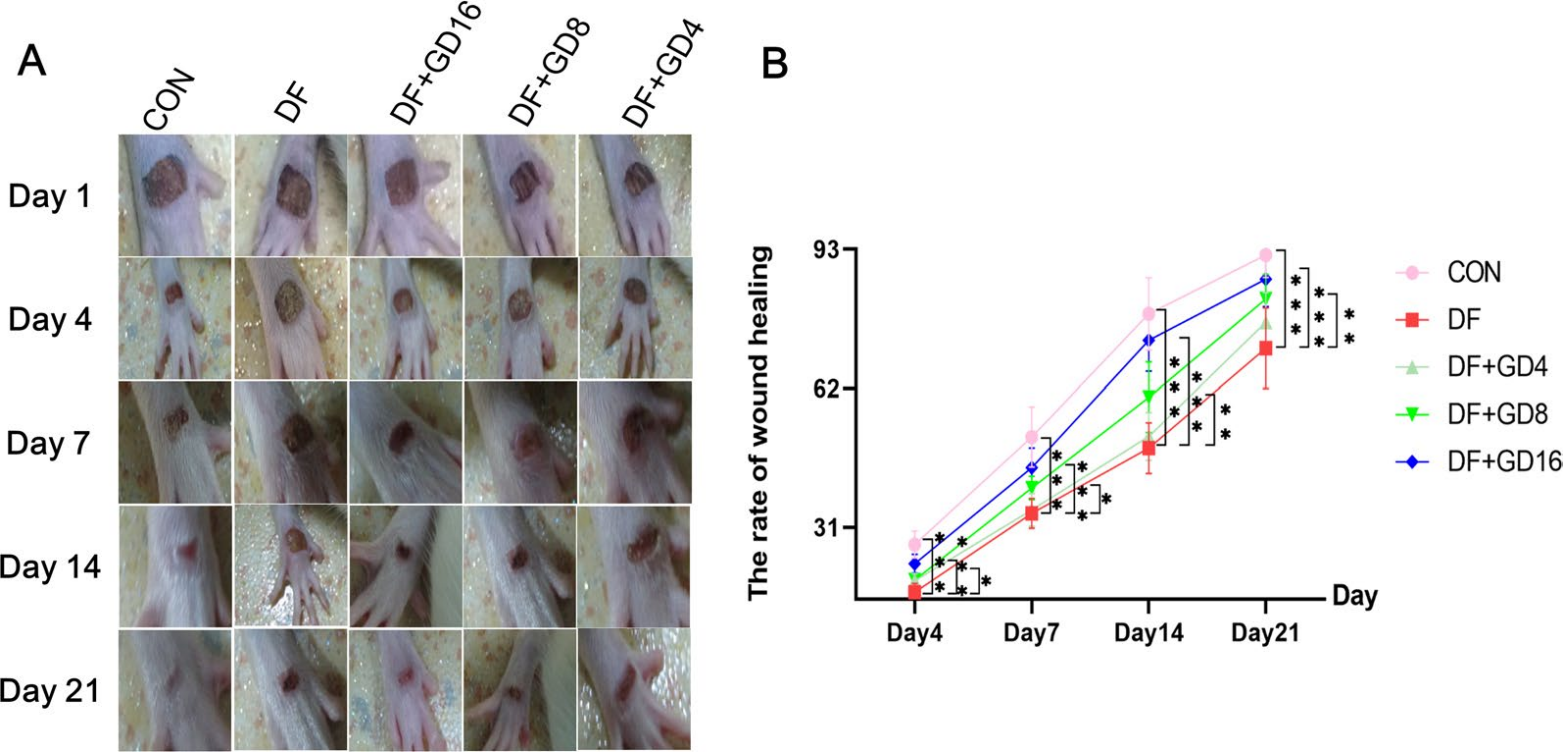
Wound healing of different time points in DF rats. **A** Images of rat foot wounds at different time points. **B** The healing rate of foot wounds at different time points in DF rats. ^*^P < 0.05, ^**^P < 0.01, ^***^P < 0.001 vs. DF

On the 1st, 4th, 7th, 14th, and 21st days after model establishment, the foot wounds of the rats were photographed for statistical analysis, and the results are shown in Fig. 8. After 21 days of gavage, compared with those in the model group, the foot wounds in the normal group were basically completely healed (*P* < 0.001). The medium and high doses of GD improved the diabetic foot wound healing rate at each time point (*P* < 0.05, vs. DF group), while the low-dose extract had no significant effect on the wound healing rate. In addition, as we expected, all dose groups of GD could reduce the blood glucose of diabetic rats (Additional file 1: Table S4).

### GD extract reduces serum levels of inflammatory factors in rats with DF

**Fig. 9:**
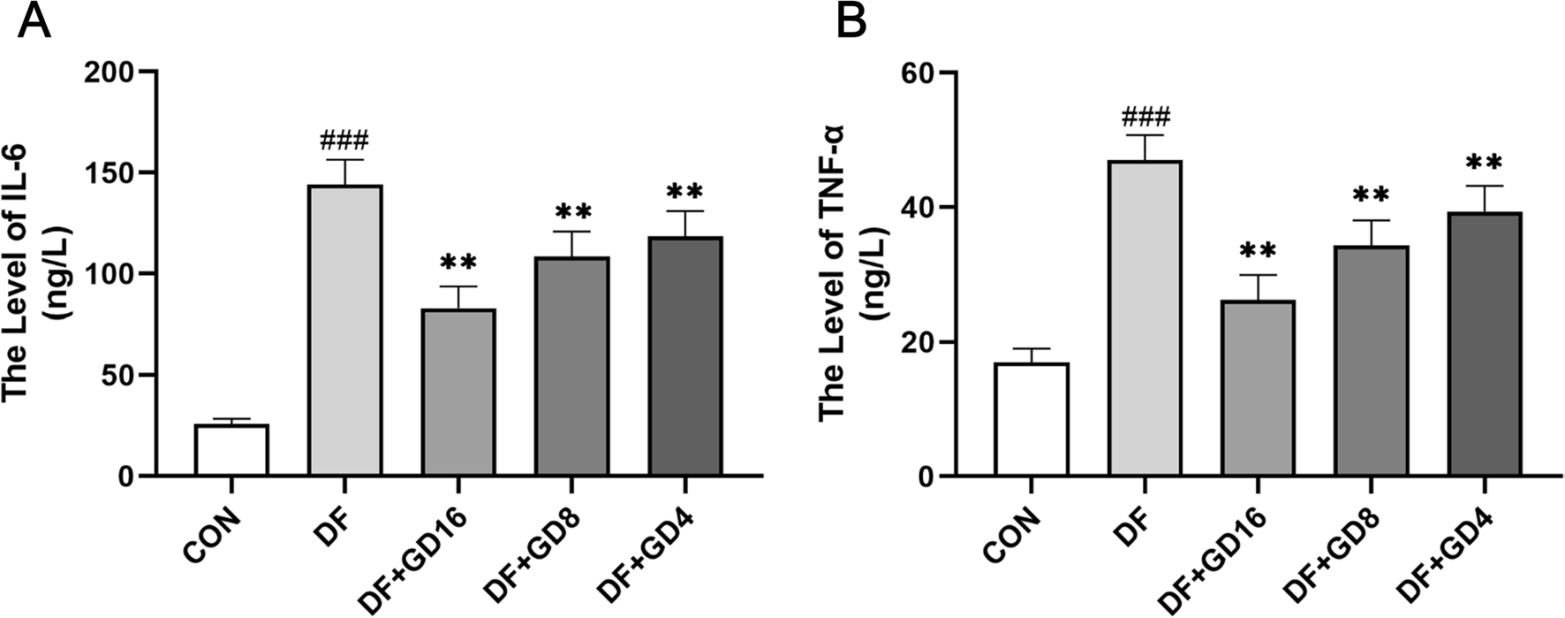
Effects of GD on Inflammatory Factors IL-1 and TNF-α in DF Rats. **A** IL-6 levels in serum. **B** TNF-α levels in serum. ^###^*P* < 0.001, vs. CON, ^**^P < 0.01, vs. DF

According to the network pharmacology results, GD may affect the levels of IL-6 and TNF-α to inhibit the occurrence and development of inflammation. Compared with those in the control group, the levels of the serum inflammatory factors IL-6 and TNF-α in the DF group were increased. Oral administration of GD significantly reduced the serum levels of IL-6 and TNF-α in the rats (Fig. 9).

### GD extract enhances the mRNA and protein expression of VEGF in rats with DF

**Fig. 10:**
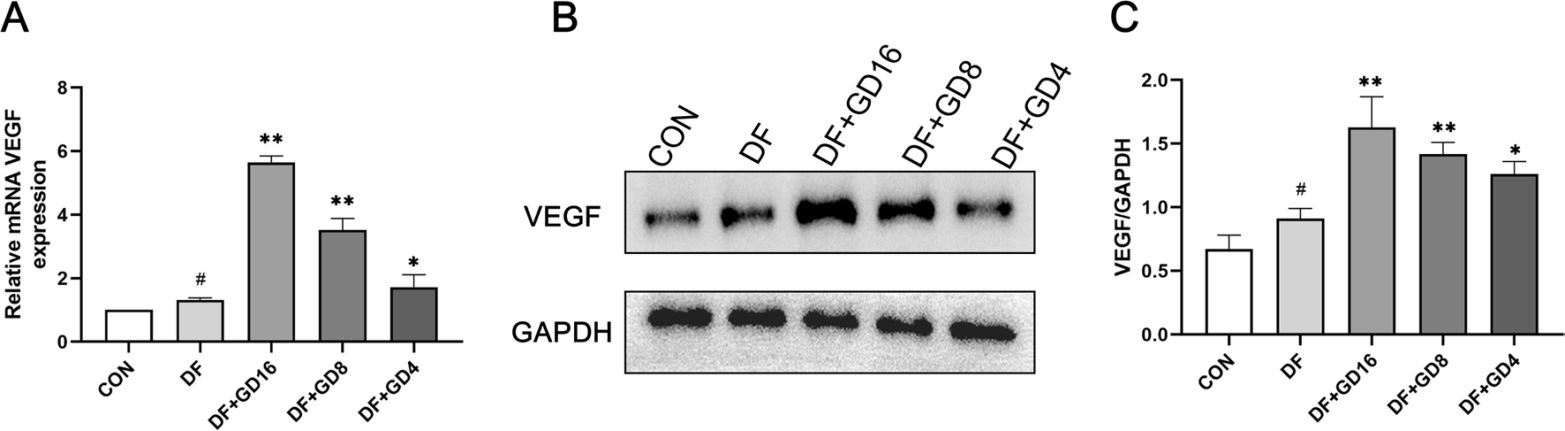
Effects of GD on the expression of VEGF mRNA and protein in lower limb ischemic tissue of DF rats. **A** The mRNA expression of VEGF. **B** Western blot results of VEGF and GAPDH. **C** Quantitative Statistics of VEGF/GAPDH. ^#^*P* < 0.05, vs. CON, ^*^P < 0.05, ^**^P < 0.01, vs. DF

Increased expression of VEGF can enhance the growth of vascular endothelium and promote the healing of ulcers. Naturally, we examined the mRNA and protein levels of VEGF. As shown in Fig. 10, compared with the control group, the mRNA and protein expression of VEGF in the DF group was increased. After treatment with GD, the expression of VEGF in the high-, medium- and low-dose GD groups was significantly increased.

## Discussion

Diabetic foot (DF) is a common complication of long-term diabetes mellitus, and it is associated with a high incidence of lower extremity ulceration and amputation (Wang et al. 2020). DF can occur for a variety of reasons and often leads to coldness, limb pain, and even ulcerative decay in both lower extremities when venous ischemia or bacterial infection occurs in the lower extremities. Currently, DF is mainly treated by anti-infection treatment and limb debridement; however, when diabetic patients have infected wounds, the wounds are large and healing is slow, which leads to a poor prognosis. Therefore, it promoting the healing of diabetic refractory skin ulcers and reducing the disability rate are hot topics in research. Several ancient prescriptions in traditional Chinese medicine have been used for the treatment of diabetic ulcers. As a hypoglycemic herbal medicine, GD has unique efficacy in treating hyperglycemia and hypertension (Li et al. 2018; Hong et al. 2020), but its efficacy in the treatment of foot ulcers complicated by diabetes as well as the underlying mechanism are not completely clear.

This study used network pharmacology methods to discover the key active ingredients of GD in the treatment of DF. Our results showed that quercetin, uridine, β-sitosterol and kaempferol are important ingredients of GD and have many targets that intersect with the disease targets of DF. Our experimental results show that GD has a good effect on the treatment of DF and can significantly improve the wound healing rate in rats with DF. Consistent with our study, Liu et al. (2021) found that quercetin inhibited podocyte apoptosis in diabetic nephropathy in vitro and in vivo by regulating the EGFR pathway. PPI network and topological analyses showed that there are 6 main targets of the active ingredients in GD. The KEGG results suggest that the AGE-RAGE signaling pathway is likely the most relevant pathway for GD. Studies have demonstrated that the specific combination of AGE and RAGE is an important mechanism that affects the level of oxidative stress in diabetic wounds (Burr et al. 2021a, b). The AGE-RAGE signaling pathway is a principal pathway in the pathogenesis of inflammatory diseases. IL6 and TNF-α are proinflammatory factors, and previous studies (Gergerlioglu et al. 2015) have revealed that IL6 and TNF-α levels are increased during infection or tissue damage, and their low expression levels are of great significance to DF wound healing (Chesworth et al. 2021). In further in vivo experiments, we measured the IL-6 and TNF-α levels in the serum of rats with DF after GD treatment. GD significantly reduced the levels of inflammatory factors in the serum of rats with DF, inhibited the progression of inflammation and promoted wound healing. Chen (Chen et al. 2020) et al. found that catalpol suppresses AGE-mediated inflammation to cure diabetic nephropathy. Similarly, Huang et al. (2019) proposed that skin injury led to increased levels of TNF-α in the peripheral blood of diabetic rats, and targeting TNF-α is a potential therapeutic option for improving diabetic wound healing. Our results align perfectly with these findings.

In addition, many studies have reported that the occurrence of DF is mainly related to inflammatory and angiogenic factors (Hotamisligil 2006; Iacobini et al.^2021^). GD significantly increased the mRNA and protein expression of vascular endothelial growth factor (VEGF). Our molecular docking results also suggest that quercetin and uridine, the active ingredients in GD, have good correlation and binding sites for the inflammatory factors IL-6 and TNF-α. Inflammatory factors cause vascular endothelial cells to promote the adhesion of leukocytes to vascular endothelial cells and release a variety of inflammatory factors, which ultimately lead to changes in cell function. Additionally, the VEGF protein can promote the growth of endothelial cells through vascular endothelial growth factor receptors, and VEGF drugs are also commonly used in the treatment of foot ulcers (Liu et al. 2022). At present, VEGF is considered to be the strongest proangiogenic factor and plays an important role in the process of wound healing (Huang et al. 2020). Under the mediation of inflammatory factors, vascular endothelial cells can promote the adhesion of leukocytes to vascular endothelial cells and release a variety of inflammatory factors, which ultimately lead to changes in cell function. Inflammatory cytokines can promote the occurrence of vascular lesions. The VEGF protein can promote the growth of endothelial cells through vascular endothelial growth factor, and VEGF drugs are also commonly used in the healing of foot ulcers. Our results showed that the mRNA and protein expression of VEGF in each GD treatment group was significantly higher than that in the rats with DF, indicating that GD could restore tissue blood supply and accelerate the healing of DF wounds. In addition, the molecular docking results of VEGF also suggest that the binding between the important components described above and VEGF is good. Many studies (Xu et al. 2015, 2020; Yi et al. 2016) have shown that GD has a good curative effect on diabetes, but few studies have been performed on its specific effect on foot complications and other complications of diabetes. This study showed that the target protein of GD in the treatment of DF was VEGF, which caused changes in the serum levels of IL-6 and TNF-α. The pathway by which these effects are mediated may be the AGE-RAGE signaling pathway.

## Conclusion

In summary, this study preliminarily explored the mechanism underlying the effects of GD on diabetic foot by means of network pharmacology and molecular docking technology. Preliminary experiments were carried out to verify that the potential mechanism by which GD functions in the treatment of DF may be related to reducing inflammation and increasing the expression of VEGF. This study provides a foundation for the further elucidation of the effective targets of drugs.

## Supplementary Information

**Additional file 1: Table S1. **RT-PCR primer sequence. **Table S2.** Herbs-Compounds list. **Table S3. **The common targets list of GD and DF. **Table S4. **Effect of GD on fasting blood glucose level of DF rats (mean ± SD, mmol/L)

## Data Availability

All data generated or analysed during this study are included in this article and its supplementary information files.

## Abbreviations

GD: *Gynura divaricata*
DF: Diabetic foot
TCMIP: Traditional Chinese medicine database platform
PPI: Protein–protein interaction
GO: Gene Ontology
KEGG: Kyoto Encyclopedia of Genes and Genomes
H-C-T: Herb-Compound-Targets
BC: Betweenness centrality
BP: Biological processes
CC: Cellular components
MF: Molecular functions
