# Supplementary material for: Exploring the mechanism by which aqueous Gynura divaricata inhibits diabetic foot based on network pharmacology, molecular docking and experimental verification
Source: Mol Med. 2023 Jan 20;29:11. doi: 10.1186/s10020-023-00605-w (PMC9862864; doi:10.1186/s10020-023-00605-w)
Supplement: Supplementary file 1 — Additional file 1: Table S1. RT-PCR primer sequence. Table S2. Herbs-Compounds list. Table S3. The common targets list of GD and DF. Table S4. Effect of GD on fasting blood glucose level of DF rats (mean ± SD, mmol/L) [file 10020_2023_605_MOESM1_ESM.docx]

Additional file 1

**Table S1 RT-PCR primer sequence**

| Gene | Forward Primer | Reverse Primer | **Product’s size** |
| --- | --- | --- | --- |
| VEGF | GCTGCTGCAATGATGAA | GCTTTGGTGAGGTTTGAT | 89 bp |
| GAPDH | TATGTCGTGGAGTCTACTGGCGTCT | AAGCAGTTGGTGGTGCAGGATG | 191 bp |

**Table S2 Herbs-Compounds list**

| Herbal medicines | Chemical compounds | MOL ID | Databse | Targets |
| --- | --- | --- | --- | --- |
| nura Divaricata | Uridine | N/A | BATMAN-TCM | TYMS, NT5C2, ADK, IMPDH1, ENPP1, POLA1, PNP, POLB, TERT, GSR, GCDH, ERO1B, IVD, NQO2, DPYD, POR, CYB5R1, MAOB, FDXR, DAO, TXNRD1, ACADS, CYB5R3, MAOA, IL4I1, AIFM1, ACOX1, ACADM, NQO1, XDH, DNMT1, ACAD8, NOS1, DLD, GFER, AK5, GUK1, ANKH, ENPP3, APRT, AK9, NUDT12, IMPDH2 |
| Gynura Divaricata | Quercetin | MOL000098 | TCMSP | PTGS1, AR, PPARG, PTGS2, HSP90AB1, PIK3CG, NCOA2, DPP4, PRSS1, TOP2A, KCNH2, SCN5A, F10, ADRB2, MMP3, PRKACA, F7, NOS3,RXRA, ACHE,GABRA1,MAOB,RELA, EPS8L2, AKT1, VEGFA, CCND1, BCL2, BCL2L1, FOS, CDKN1A, EIF6, BAX, CASP9, PLAU, MMP2, MMP9, MAPK1, IL10, EGF, RB1, TNF, IL6, AHSA1, CASP3, TP53, ELK1, NFKBIA, POR, ODC1, XDH, CASP8, TOP1, RAF1, SOD1, PRKCA, MMP1, HIF1A, STAT1, RUNX1T1, CDC2, HEL-S-89n, ERBB2, PPARG, ACACA, HMOX1, CYP3A4, CYP1A2, CAV1, MYC, F3, GJA1, CYP1A2, ICAM1, IL1B, CCL2 , SELE, VCAM1, PTGER3, CXCL8, PRKCB, BIRC5, DUOX2, NOS3, HSPB1, TGFB1, MGAM, IL2, NR1I2, CYP1B1, CCNB1, PLAT, THBD, SERPINE1, COL1A1, IFNG, ALOX5AP, PTEN, IL1A, MPO, TOP2A, NCF1, ABCG2, HAS2, GSTP1, NFE2L2, NQO1, PARP1, AHR, PSMD3, SLC2A4, COL3A1, CXCL11, CXCL2, DCAF5, NR1I3, CHEK2, INSR, CLDN4, PPARA, PPARD, HSF1, CRP, CXCL10, CHUK, SPP1, RUNX2, RASSF1, E2F1, E2F2, ACP3, CTSD, IGFBP3, IGF2, CD40LG, IRF1, ERBB3, PON1, DIO1, PCOLCE, NPEPPS, HK2, NKX3-1, RASA1, GSTM1, GSTM2 |
| Gynura Divaricata | Kaempferol | MOL000422 | TCMSP | UGT3A1, CRYZ, VKORC1, NQO1, PTGS1, AR, PPARG, PTGS2, HSP90, PIK3CG, NCOA2, PRKACA, DPP4, PGR, CHRM1, NOS3, ACHE, GABRA2, CHRM2, ADRA1B, GABRA1, TOP2A, CAMKK2, RELA, BCL2, BAX, TNF, CASP3, AHSA1, XDH, MMP1, STAT1, PPARG, HMOX1, CYP3A4, CYP1A2, CYP1A1, ICAM1, SELE, VCAM1, CYP1B1, NR1I2, HAS2, AHR, GSTP1, PSMD3, SLC2A4, INSR, DIO1, PP3CA, GSTM1, GSTM2, AKR1C3, SLPI |
| Gynura Divaricata | Nicotinic acid | MOL010492 | TCMSP | GABRA1,GABRA2 |
| Gynura Divaricata | Sucrose | MOL000842 | TCMSP | FOS, FOSL2, PPARG, CYP1A2, COL3A1, NR4A1, ALAD, LCT, , AMY2, |
| Gynura Divaricata | beta-sitosterol | MOL000358 | TCMSP | PGR, NCOA2, PTGS1, PTGS2, HSP90AB1, PIK3CG, KCNH, PRKACA, DRD1, CHRM3, CHRM1 , SCN5A, GABRA2 CHRM4 , PDE3A, HTR2A, GABRA5, ADRA1A, GABRA3, CHRM2, ADRA1B, ADRB2, CHRNA2, SLC6A4, OPRM1, GABRA1, CHRNA7, BCL2, BAX, CASP9, CASP3, CASP8, PRKCA, TGFB1, PON1, MAP2 |
| Gynura Divaricata | Adenosine | MOL001787 | TCMSP | PTGS1, PNP,PTGS2, PTPN1, ADORA2A, ADAL |
| Gynura Divaricata | Dibutyl terephthalate | MOL011366 | TCMSP | CHRM1, ADRB2 |
| Gynura Divaricata | Daucosterol | MOL000093 | TCMSP | N/A |
| Gynura Divaricata | Methyl chlorogenate | MOL003048 | TCMSP | N/A |
| Gynura Divaricata | beta-D-fructofuranose | MOL007789 | TCMSP | N/A |
| Gynura Divaricata | Nystose | MOL009535 | TCMSP | N/A |
| Gynura Divaricata | 1F-Fructofuranosylnystose | MOL009510 | TCMSP | N/A |
| Gynura Divaricata | Heriguard | MOL001955 | TCMSP | N/A |
| Gynura Divaricata | 3, 4-Dicaffeoylquinic acid | MOL003067 | TCMSP | N/A |
| Gynura Divaricata | 3,5-o-Dicaffeoylquinic acid | MOL010033 | TCMSP | N/A |
| Gynura Divaricata | 4,5-Dicaffeoylquinic acid | MOL003068 | TCMSP | N/A |
| Gynura Divaricata | stigmasterol-5-O- beta-D-glucoside | N/A | TCMSP | N/A |
| Gynura Divaricata | 5-hydroxy-picolinic acid | N/A | TCMSP | N/A |
| Gynura Divaricata | Methyl-5-hydroxy-2- pyridinecarboxylate | N/A | TCMSP | N/A |
| Gynura Divaricata | 1-kestose | N/A | TCMSP | N/A |
| Gynura Divaricata | pedatisectine G | N/A | TCMSP | N/A |
| Gynura Divaricata | 5-hydroxypyridine-2-carboxylic acid methyl ester | N/A | TCMSP | N/A |
| Gynura Divaricata | 2-(1', 2', 3', 4'-tetrahydroxybutyl)-6-(2", 3", 4"-trihydroxybutyl) | N/A | TCMSP | N/A |
| Gynura Divaricata | 2-(1', 2', 3', 4'-tetrahydroxybutyl)-5-(2", 3", 4"-trihydroxybutyl) -pyrazine | N/A | TCMSP | N/A |

Filter Conditions: TCMSP: OB≥30, DL≥0.18. BATMAN-TCM: Score cutoff≥20, *P*<0.05.

**Table S3. The common targets list of GD and DF**

| PTGS1 | ACHE | MMP9 | TOP1 | GJA1 | CYP1B1 | NFE2L2 | E2F1 | HTR2A | IMPDH1 |
| --- | --- | --- | --- | --- | --- | --- | --- | --- | --- |
| AR | GABRA1 | MAPK1 | RAF1 | ICAM1 | CCNB1 | PARP1 | CTSD | GABRA5 | TERT |
| PPARG | MAOB | IL10 | SOD1 | IL1B | PLAT | SLC2A4 | IGFBP3 | ADRA1A | MAOA |
| PTGS2 | RELA | EGF | PRKCA | CCL2 | THBD | COL3A1 | IGF2 | SLC6A4 | ENPP1 |
| DPP4 | AKT1 | RB1 | MMP1 | SELE | SERPINE1 | DCAF5 | CD40LG | OPRM1 | GSR |
| KCNH2 | VEGFA | TNF | HIF1A | VCAM1 | COL1A1 | NR1I3 | IRF1 | CHRNA7 | DPYD |
| SCN5A | CCND1 | IL6 | STAT1 | CXCL8 | IFNG | CHEK2 | ERBB3 | MAP2 | AIFM1 |
| F10 | BCL2 | CASP3 | ERBB2 | PRKCB | ALOX5AP | INSR | PON1 | TYMS | DNMT1 |
| ADRB2 | BCL2L1 | TP53 | HMOX1 | BIRC5 | PTEN | CLDN4 | GSTM1 | GCDH | ACOX1 |
| MMP3 | FOS | NFKBIA | CYP3A4 | HSPB1 | IL1A | PPARA | DRD1 | NT5C2 | NOS1 |
| PRKACA | CDKN1A | POR | CYP1A2 | TGFB1 | MPO | CRP | CHRM3 | ACADS | ANKH |
| F7 | BAX | ODC1 | CAV1 | MGAM | NCF1 | CXCL10 | CHRM1 | ADK | GFER |
| NOS3 | CASP9 | XDH | MYC | IL2 | ABCG2 | SPP1 | GABRA2 | POLB | IMPDH2 |
| RXRA | PLAU | CASP8 | F3 | NR1I2 | GSTP1 | RUNX2 | PDE3A | IVD | MMP2 |

**Table S4. Effect of GD on fasting blood glucose level of DF rats (**mean ± SD, mmol/L**)**

| Group | 0d | 4d | 7d | 14d | 21d |
| --- | --- | --- | --- | --- | --- |
| CON | 5.88 ± 0.44^***^ | 5.32 ± 0.51^***^ | 5.03 ± 0.44^**^^*^ | 5.70 ± 0.51^***^ | 5.26 ± 0.52^***^ |
| DF | 26.39 ± 3.41 | 26.36 ± 3.48 | 27.82 ± 1.71 | 27.55 ± 1.34 | 26.47 ± 1.67 |
| DF+GD16 | 26.28 ± 4.10 | 25.16 ± 3.46 | 24.46 ± 1.72^**^ | 23.72 ± 2.00^**^ | 20.42 ± 2.49^***^ |
| DF+GD8 | 26.47 ± 3.59 | 25.43 ± 0.51 | 24.93 ± 1.22^*^ | 24.06 ± 1.56^*^ | 21.87 ± 1.67^**^ |
| DF+GD4 | 26.73 ± 3.50 | 25.84 ± 3.83 | 25.26 ± 1.87^*^ | 24.07 ± 2.39^*^ | 22.52 ± 1.78^**^ |

^*^*P*<0.05, ^**^*P*<0.01, ^***^*P*<0.001 vs. DF.
